# Outcomes reported in trials of children and adolescent knee injuries: a systematic review

**DOI:** 10.1302/2633-1462.68.BJO-2025-0005.R1

**Published:** 2025-08-21

**Authors:** Ignatius Liew, Wen Xian Low, Adeel Ikram, Stephen McDonnell, Ben Arthur Marson, Core Kids Knee Steering committee

**Affiliations:** 1 Queen Mary University of London, London, UK; 2 Department of Academic Orthopaedics, Trauma and Sports Medicine. Academic Unit of Injury, Recovery and Inflammation Sciences, The University of Nottingham, Nottingham, UK; 3 NIHR Nottingham Biomedical Research Centre, Nottingham, UK; 4 Department of Orthopaedics and Trauma, Academic Unit of Injury, Recovery and Inflammation Sciences, Queens Medical Centre, Nottingham, UK; 5 Musculoskeletal Surgery, Inflammation and Recovery Theme NIHR Biomedical Research Centre, University of Nottingham, Queens Medical Centre, Nottingham, UK; 6 Department of Trauma and Orthopaedics, Addenbrooke’s Hospital, Cambridge University Hospital, Cambridge, UK; 1 The University of Nottingham, Nottingham Biomedical Research Centre, Queens Medical Centre Nottingham, Nottingham, UK; 2 University of Cambridge, Cambridge, UK; 3 Musculoskeletal, Surgery, Inflammation and Recovery Theme NIHR Biomedical Research Centre University of Nottingham, Nottingham, UK; 4 St George's Hospital, London, UK; 5 Cambridge University Hospital NHS Foundation Trust, Cambridge, UK; 6 School of Human Sciences (Exercise and Sport Science), University of Western Australia, Perth, Australia; 7 Nottingham University Hospitals NHS Trust, Nottingham, UK; 8 Birmingham Children's Hospital, Birmingham, UK; 9 Ann & Robert H. Lurie Children's Hospital of Chicago, Chicago, Illinois, USA; 10 Sheffield Children's Hospital, Sheffield, UK; 11 Medical School, Division of Surgery, University of Western Australia and Orthopaedic Research Foundation of Western Australia, Perth, Australia; 12 Broomfield Hospital, Chelmsford, UK; 13 Warwick Medical School, University of Warwick, Warwick, UK; 14 Bart’s and the London School of Medicine, Queen Mary University of London, London, UK; 15 Cambridge University Hospital NHS Foundation Trust, Cambridge, UK

**Keywords:** Knee, Kids knee, Kids knee injuries, Paediatrics, ACL, Patellofemoral dislocation, Meniscus, OCD, knee injuries, patient-reported outcome measures (PROMs), IKDC, International Knee Documentation Committee, clinical trials, lower limb, MEDLINE, Knee injury and Osteoarthritis Outcome Score; KOOS, patient-reported outcomes measurement information system

## Abstract

**Aims:**

To systematically review published evidence of outcomes reported in trials of knee injuries in children and adolescents.

**Methods:**

We searched the following databases from inception to 29 July 2024: OVID MEDLINE, Embase, Cochrane CENTRAL, Clinicaltrials.gov, and the World Health Organization (WHO) International Clinical Trials Registry Platform (ICTRP). In total, 13,146 studies were identified; after removing duplicates, 9,796 studies were yielded for screening following PRISMA guidelines. Data extraction was performed by two researchers, and 15 trials were included in the final analysis. Outcomes reported by trials were mapped to the domains within the WHO International Classification of Function framework (ICF), comprising four main categories: Body functions (b), Activities and participation (d), Environmental factors (e), and Body structure (s).

**Results:**

A total of 83 outcomes were identified from 15 trials, representing 35 WHO domains. The most common domain reported mapped to the ICF framework was structure of the lower limb (s750; 93.3%), followed by sensation of pain (b280; 86.7%), mobility of joint function (b710; 86.7%), and function of the joints and bones (b729; 86.7%). Patient satisfaction was reported in two trials (13.3%) trials. Primary outcomes were not reported in seven trials (46.6%). Pedi-International Knee Documentation Committee (IKDC) was the most common patient-reported outcome measure in seven trials (46.6%).

**Conclusion:**

Outcome measure tools reported in children and adolescent knee injuries are highly variable and inconsistent. Currently, there are no core outcome sets (COS) for these injuries, highlighting an urgent need to improve standardization and consistency in trial reporting. A key recommendation for the COS development is accounting for pathology-specific subcategories, given the difference in emphasis on WHO ICF domains across various knee injuries. In the future, these approaches will ensure the COS has comprehensive yet unique priorities for each condition.

Cite this article: *Bone Jt Open* 2025;6(8):971–982.

## Introduction

The incidence of paediatric anterior cruciate ligament (ACL) injury is increasing, especially in adolescent athletes and those who are skeletally immature, with some studies describing a three-fold increase and up to 55% increase in girls over the last two decades.^1-6^ Soft-tissue knee injuries share the same increasing incidence, either in isolation or often in association with each other due to the mechanism of injury.^7^ These include meniscal tears, patellofemoral pathologies such as dislocation, osteochondral dissecans, and fractures.^7,8^ There is a sex disparity, with an eight-fold increase in ACL injuries disproportionately affecting female athletes more than males.^9-11^ Some examples illustrated in this review article summarize them to anatomical differences, biomechanics (congenital valgus knee angle), and hormonal factors, as well as the availability of training and prevention programmes.^12^ Variability exists within pathways for acute soft-tissue knee injuries that can lead to delay in accurate diagnoses.^13^ Delays in ACL reconstruction are associated with medial chondral injuries,^14^ in which the prevalence of osteoarthritis increases over time, of up to 51.6% at 20 years since the index ACL injury, and a three- to six-fold increase in the risk of the injured knee.^1,15-17^

Outcomes for soft-tissue knee injuries are among the National Institute for Health and Care Research (NIHR) James Lind Alliance’s top ten priorities among ages 12 to 17 years through the Priority Setting Partnerships (PSP).^1^ Before trials can be delivered to compare treatments, we need to know the most relevant and valid outcomes to measure.^18-21^ Unfortunately, there is significant heterogeneity in reporting outcomes for paediatric knee injuries, which limits our ability to draw conclusions and compare study outcomes in systematic reviews.^22^

Core outcome sets (COSs) are increasingly being used within musculoskeletal and orthopaedic research. However, they do not exist for children and adolescent knee injuries. As a minimum, a COS is required in trials to consistently measure several broad domains. While providing an agreed set of outcomes, trialists and methodologists can compare treatment outcomes, reducing heterogeneity. With COS, we can reduce research waste, produce high-quality meta-analyses internationally, and reduce the risk of inappropriate trial studies that limit the clinical value.^18,19,23,24^

The Core Kids Knee Steering Committee, consisting of key stakeholders and an international faculty, will aim to define the current outcomes reported in trials of children and adolescent knee injuries, with the principles outlined as the first part of the methodology within the COMET initiative.^24,25^ No previous studies have attempted to define the outcome domains. The outcome domains are analyzed and grouped according to the World Health Organization (WHO) International Classification of Functioning, Disability and Health framework (ICF).^19,21,26,27^ The outcome tools used were evaluated to report the existing patient-reported outcome measures (PROMs) and surgeon- and patient-reported outcomes, defined as instruments or questionnaires to measure patient-reported outcomes (PROs).^28^

## Methods

The COMET initiative methodology and PRISMA guidelines were used to identify and classify reported outcomes in clinical trials.^24,29^ The systematic review was prospectively registered with the PROSPERO database CRD 42024554304,^30^ summarizing the inclusion criteria and protocol. The study protocol as part of the COMET initiative methodology will be available in a separate publication. We included randomized and quasi-randomized controlled trials with no data restrictions. Trial participants involved children and adolescents, which included patients aged five to 16 years. Patients sustaining a knee injury (patellofemoral dislocation, ACL injury or rupture, meniscal and cartilage pathologies, among others) were included.

The following electronic databases were searched from inception to 29 July 2024: OVID MEDLINE, Embase, Cochrane Central Register of Controlled Trial (CENTRAL), Clinicaltrials.gov, and the WHO International Clinical Trials Registry Platform (ICTRP). The search strategy is included in the Supplementary Material. We included and devised the search strategy to identify knee injuries but excluded lower limb fractures due to existing COS.^18^ A previously validated search filter for child health and randomized studies was used for each database.^31,32^ Further studies were retrieved from manual searches of Clinicaltrials.gov and WHO ICTRP, completed on 29 July 2024, in line with Cochrane guidelines to identify active studies, relevant unpublished studies, trials, and protocols.^33^

Two independent researchers (IL, WXL) screened the titles and abstracts for potential full-text articles. If a consensus was not reached, a final vote was resolved by the senior author (BAM) regarding article inclusions. The data extraction and classification were performed independently into the second-level WHO ICF domains, independently by IL and WXL, with discrepancies resolved by consensus with the senior author (BAM).

The risk of bias and study quality assessments were not performed as part of this review, given that this was not the aim of the review, in line with previous studies of similar methodology.^18^ The characteristics of each trial, including outcome measures reported, injuries included, and participant age, were evaluated. A narrative synthesis approach was employed to report trial outcomes, incorporating verbatim outcome descriptions and quantification of the frequency with which each outcome was reported. Subgroup analyses were performed on subcategories/injuries with at least three trials.

We mapped each identified outcome measure to the WHO ICF framework, previously demonstrated as the analysis framework for many children’s orthopaedic COS.^18,26,34^ The framework has been shown internationally as the standard for measuring outcomes, which included the following: 1) body function (b); 2) activity and participation (d); 3) body structure (s); and 4) environmental factors (e). The number of trials assessing each ICF domain, as well as the number of trials in which each domain was the primary outcome, were recorded and expressed as percentages of the total number of trials.

The number of trials assessing each ICF domain, as well as the number of trials in which each domain was the primary outcome, were recorded and expressed as percentages of the total number of trials.

### Patient demographic data

**Fig. 1:**
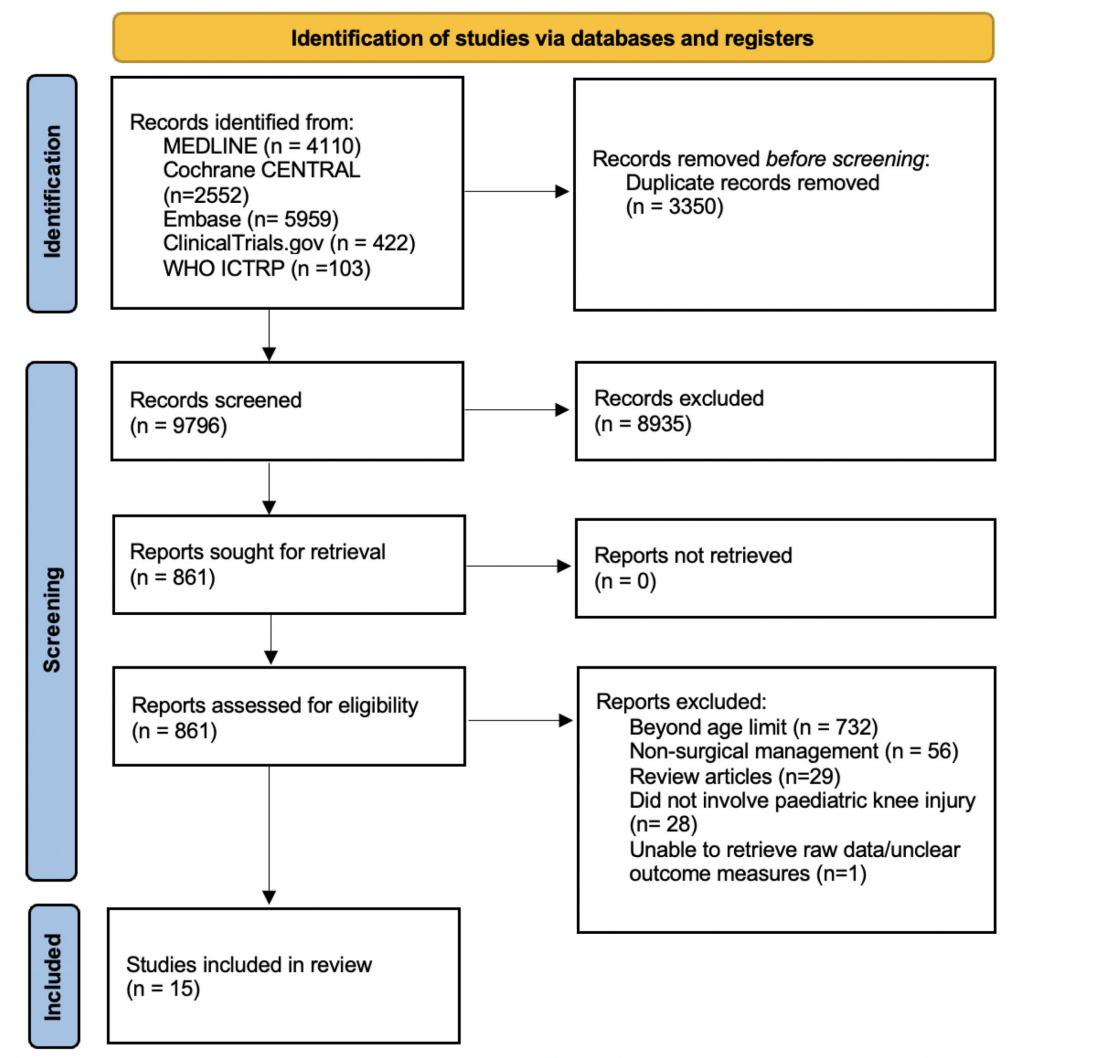
PRISMA flow diagram, which included searches of the following databases and registers: seven completed and published trials, six active trials and two trials not yet recruiting. WHO ICTPR, World Health Organization International Clinical Trials Registry Platform.

A total of 13,146 studies yielded from our searches were screened following PRISMA guidelines (Figure 1). After removing duplicates, 9,796 studies were screened with full-text screening of 861 articles. From this, 15 studies were eligible and included in the analysis.

A total of 83 distinct WHO ICF outcomes were reported, with a median number of outcomes reported per trial of five (IQR 3 to 6). These distinct outcomes were reported and mapped onto second-level outcome domains across the ICF framework. The following number of domains were identified: 18 activity and participation (d), 15 body function (b), one environmental (e), and one body structural (s). Complication rates were reported in seven trials (46.6%), as well as the reoperation rate in five trials (33.3%): these outcomes could not be mapped onto the ICF framework.

The study demographic details are summarized in Table I. 

**Table I.** Demographic details of studies.

Study	Country	Condition of interest	Sites, n	Participants or enrolment estimates, n	Mean age, or age for inclusion, yrs	Status
Ebert (2024)^35^	Australia	ACL tear	1	44	8 to 17	Not yet recruiting
Bade et al (2022)^36^	Australia	ACL tear	1	100	10 to 18	Recruiting
Albright (2021)^37^	USA	ACL tear	4	160	12 to 18	Recruiting
Kass (2021)^38^	USA	ACL tear	1	240	0 to 18	Recruiting
NCT03896464 (2019)^39^	Canada	ACL tear	3	100	10 to 18	Recruiting
NCT04039971 (2019)^40^	USA	ACL tear	1	150	12 to 19	Recruiting
Askenberger et al (2018)^41^	Sweden	Primary patellar dislocation	1	74	13.1 (9 to 14, SD 1.1)	Completed and published
Regalado et al (2016)^42^	Finland	Primary patellar dislocation	NR	36	13.5 (8 to 16)	Completed and published
Palmu et al (2008)^43^	Finland	Primary patellar dislocation	1	62	13 (9 to 15, SD 2)	Completed and published
Matuszewski et al (2018)^44^	Poland	Recurrent patellar dislocation	2	44	14.9 (13 to 17)	Completed and published
Zhao et al (2012)^45^	China	Recurrent patellar dislocation	1	60	14.9 (12 to 19, SD 1.45)	Completed and published
Heyworth et al (2023)^46^	USA	Osteochondritis dessicans	14	91	12.3 (9 to 15, SD 1.32)	Completed and published
NCT02397278 (2015)^47^	USA	Osteochondritis dessicans	1	15	11.6 (10 to 14)	Completed, not published
Kang (2017)^48^	China	Osteochondral fracture after patellar dislocation	1	72	9 to 17	Not yet recruiting
NCT06176183 (2021)^49^	Lithuania	Meniscal tear	2	100	0 to 19	Recruiting

ACL, anterior cruciate ligament.

## Results

Outcomes are reported stratified by types of knee injury in Table II. The frequency and distribution of ICF framework outcome domains are summarized in Table III. The most common domain reported mapped to the ICF framework was structure of lower limb (s750; 93.3%), followed by sensation of pain (b280; 86.7%), mobility of joint function (b710; 86.7%), and function of the joints and bones (b729; 86.7%). Patient satisfaction was reported in two trials (13.3%), with only one trial offering a binary response option: satisfied and unsatisfied.

**Table II.** Outcomes reported stratified by types of knee injury.

Type of knee injury	N	Mean age (range)/age for inclusion	PROMs	Other outcomes
ACL	6	0 to 19	Pedi-IKDC Lysholm HSS Pedi-FABS KOOS-Child Marx activity rating scale Tegner activity scale PROMIS Paediatric Depressive Symptoms – Short Form 8 a PROMIS Paediatric Anxiety – Short Form 8 a PROMIS Paediatric Physical Activity – Short Form 8 a PROMIS Paediatric Mobility – Short Form 7 a	**Subjective measures** Pain Functionality Healing Quality of life **Objective measures** Knee pain Knee range of motion Anteroposterior stability Rotational stability Thigh muscle strength Tibial internal and external rotation strength Balance Forward step-down test Single leg hop **Adverse events** Postoperative arthrofibrosis Complication rates Growth disturbance Lower limb deformity Knee reinjury rate ACL re-rupture rate Distal femoral and/or proximal tibial/fibular physeal injury Reoperation rate **Radiological** Leg length deformity Angular deformity Muscle-tendon morphology Graft morphology **Others** Return to sport rate Time to return to sport Length of return to sports Procedure time
Acute patella dislocation	3	13.15 (8 to 16)	KOOS-Child Hughston VAS EQ-5D-Y Kujala score Tegner Activity scale	**Subjective measures** Pain Patient satisfaction Knee function Subjective grade **Objective measures** Rate of positive apprehension test Knee range of motion Presence of patellar tilt on clinical exam Thigh circumference Thigh muscle strength Thigh muscle strength symmetry **Adverse events** Complication rate Redislocation rate Time from first lateral patellar dislocation to recurrence Activity involved during redislocation Reoperation rate **Radiological** MRI assessment for trochlear dysplasia, patellar alta, lateral patellar tilt, elevated tibial tubercle-trochlear groove
Recurrent patella dislocation	2	14.9 (12 to 19)	IKDC Kujala Lysholm score Tegner score	**Subjective measures** Subjective symptom rating (not otherwise specified) **Objective measures** Rate of positive apprehension test Rate of lateral patellar translation grade Rate of firm endpoint in lateral translation **Adverse events** Complication rate Patellar redislocation rate Patellar instability rate **Radiological** Ultrasound examination for MPFL graft tension and patella tracking Radiological assessment for patellar tilt angle CT assessment for congruence angle, lateral patellar angle, and patellar tilt angle **Others** Rate of return to activity Median duration of operation
Osteochondritis dissecans	3	12.2 (9 to 15)	Pedi-IKDC Lysholm score KOOS score KOOS QOL subdomain score Marx Activity Scale	**Adverse events** Complications Reoperation rate **Radiological** Radiological assessment for healing MRI assessment for cartilage healing **Others** Return to sport rate Tourniquet time fluoroscopy time Mean number of K-wire passes Length of K-wire Rate of additional drilling through intercondylar notch
Osteochondral fracture after patellar dislocation	1	9 to 17	IKDC Lysholm score	**Subjective measures** Pain Patient satisfaction **Objective measures** Range of motion **Adverse events** Patellar redislocation rate Infection rate Knee stiffness **Radiological** MRI assessment **Others** Return to sports rate
Meniscal tear	1	0 to 19	Pedi-IKDC Lysholm PedsQL	**Radiological** MRI assessment

ACL, anterior cruciate ligament; EQ-5D-Y, EuroQol five-dimension questionnaire-youth; IKDC, International Knee Documentation Committee; KOOS, Knee injury and Osteoarthritis Outcome Score; MPFL, medial patellofemoral ligament; HSS Pedi-FABS, Hospital for Special Surgery Paediatric Functional Activity Brief Scale; Pedi-IKDC, Paediatric International Knee Documentation Committee; PedsQL, Paediatric Quality of Life; PROMIS, patient-reported outcomes measurement information system; PROMs, patient-reported outcome measures; QoL, quality of life; VAS, visual analogue scale.

**Table III.** Outcomes mapped to World Health Organization International Classification of Functioning, Disability and Health framework.

ICF outcome domain	Trials, n (%)	ICF outcome domain	Trials, n (%)
Body function b280 Sensation of pain b710 Mobility of joint functions b729 Function of the joints and bones, other specified and unspecified b789 Movement function b152 Emotional functions b715 Stability of joint function b770 Gait pattern functions b134 Sleep functions b730 Muscle power functions b740 Muscle endurance functions b130 Energy and drive functions b144 Memory function b435 Immunological system functions b780 Sensations related to muscles and movement functions b820 Repair functions of the skin	13 (86.7) 13 (86.7) 13 (86.7) 11 (73.3) 10 (66.7) 10 (66.7) 8 (53.3) 3 (20) 3 (20) 2 (13.3) 1 (6.7) 1 (6.7) 1 (6.7) 1 (6.7) 1 (6.7)	Activity and participation d410 Changing basic body position D451 Going up and down stairs d455 Moving around d299 General tasks and demands, other d920 Recreation and leisure d450 Walking d465 Moving around using equipment d415 Maintaining a body position d430 Lifting and carrying objects d510 Washing oneself d640 Doing housework d470 Using transportation d298 General tasks and demands, other specified d530 Toileting d540 Dressing d620 Acquisition of goods and services d750 Informal social relationships d820 School education	12 (80) 12 (80) 12 (80) 11 (73.3) 10 (66.7) 8 (53.3) 6 (40) 5 (33.3) 5 (33.3) 5 (33.3) 5 (33.3) 5 (33.3) 4 (26.7) 1 (6.7) 1 (6.7) 1 (6.7) 1 (6.7) 1 (6.7)
Body structure s750 Structure of lower limb	14 (93.3)		
Environmental factors e320 Friends	4 (26.7)		

ICF, International Classification of Functioning, Disability and Health framework.

Table IV summarizes the distribution of outcome domains mapped against WHO ICF of primary outcomes reported in these trials. Structure of lower limb (s750) was the most common primary outcome in six trials (40.0%). Five trials (33.3%) reported these based on clinical findings: three of these (60%) were graft failure rate/re-rupture, one (20%) re-dislocation rate and one (20%) anteroposterior (AP) laxity of the knee measured on KT-1000. One trial reported radiological (MRI) outcomes to assess cartilage healing using dGEMRIC MRI. PROMs were specified as the primary outcome in two trials (13.3%) and visual analogue score (VAS) for knee pain was used in three trials (20%). Primary outcomes were not specified in seven trials (46.6%).

**Table IV.** Distribution of primary outcome mapped against World Health Organization International Classification of Functioning, Disability and Health framework outcome domains.

Primary outcome domain or score	Trials, n (%)
s750 Structure of lower limb	6 (40)
Not reported or stated	5 (33.3)
d920 Recreation and leisure	2 (13.3)
b710 Mobility of joint functions	1 (6.7)
b780 Sensations related to muscles and movement functions	1 (6.7)
Rate of reoperation	Not mappable
Functional outcome score: Lysholm	2 (13.3)
Functional outcome score: IKDC	1 (6.7)
Functional outcome score: Pedi-IKDC	1 (6.7)
Functional outcome score: PedsQL	1 (6.7)

IKDC, International Knee Documentation Committee; Pedi-IKDC, Paediatric International Knee Documentation Committee; PedsQL, paediatric quality of life.

**Fig. 2:**
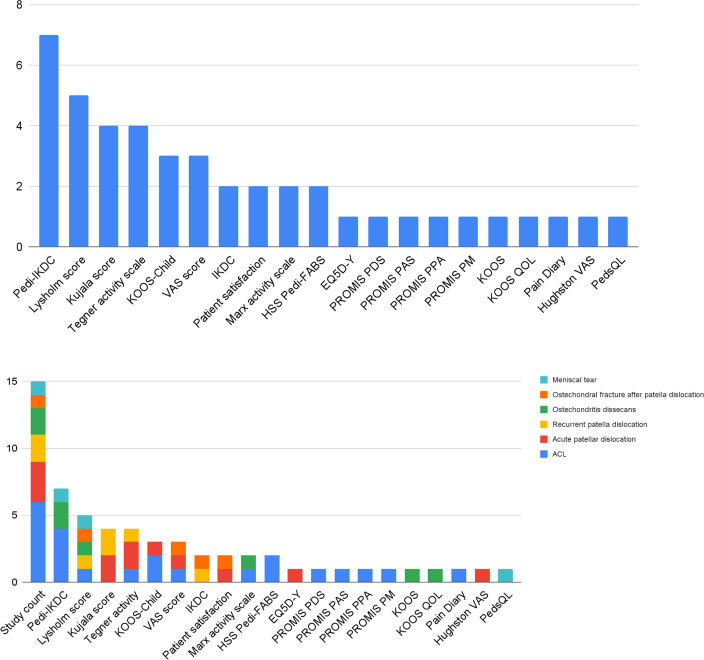
Patient-reported outcome measures (PROMs) used in trials. ACL, anterior cruciate ligament; EQ5D-Y, EuroQol-five-dimension questionnaire-Youth; HSS Pedi-FABS, the Hospital for Special Surgery Paediatric Functional Activity Brief Scale; Hughston VAS, Hughston visual analogue scale; IKDC, International Knee Documentation Committee; KOOS-Child, Knee injury and Osteoarthritis Outcome Score for Children; KOOS, Knee Injury and Osteoarthritis Outcome Score; KOOS QOL, Knee injury and Osteoarthritis Outcome Score quality of life subdomain; Pedi-IKDC, Paediatric International Knee Documentation Committee; PedsQL, paediatric quality of life; PROMIS PA-8a, patient-reported outcomes measurement information system paediatric anxiety short form 8 a; PROMIS PDS-8a, patient-reported outcomes measurement information system paediatric depressive symptoms short form 8 a; PROMIS PM-7a, patient-reported outcomes measurement information system paediatric mobility short form 7 a; PROMIS PPA-8a, patient-reported outcomes measurement information system paediatric physical activity short form 8 a.

There were 20 outcome tools identified, with all outcome measures classified as patient- or proxy-reported outcome measures (PROMs) summarized in Table V. The most frequently used PROM was Pedi-IKDC seven (46.6%), Lysholm score five (33.3%), Kujala score four (26.7%), and Tegner activity scale four (26.7%) (Figure 2). When sub-categorized based on primary pathology, the Kujala score was used in four trials (80%) in patella-related pathologies, with Pedi-IKDC used in ACL pathology in four (66.7%) trials, followed by osteochondritis dissecans (OCD) two (100%) and meniscal tear one (100%).

**Table V.** Outcome instruments in trials of children and adolescent knee injuries - patient- or proxy-reported outcome measures (PROMs). No surgeon-/clinician-reported outcome measures (SROMs) were identified.

Outcome instrument	Type of tool	Number of trials	Items	Original construct		
				Physical function	QoL	Emotional function
Pedi-IKDC	PROM	7	21	X	X	
Lysholm score	PROM	5	8	X		
Kujala score	PROM	4	12	X		
Tegner activity scale	PROM	4	1	X		
KOOS-Child	PROM	3	39	X	X	X
VAS score	PROM	3	1		X	
IKDC	PROM	2	19		X	
Patient satisfaction	PROM	2	4		X	
Marx activity scale	PROM	2	6	X		
HSS Pedi-FABS	PROM	2	8	X		
EQ-5D-Y	PROM	1	6	X	X	X
PROMIS PDS-8a	PROM	1	8		X	X
PROMIS PA-8a	PROM	1	8		X	X
PROMIS PPA-8a	PROM	1	8	X		
PROMIS PM-7a	PROM	1	7	X		
KOOS	PROM	1	40	X	X	X
KOOS QoL	PROM	1	4		X	
Pain diary	PROM	1	1		X	
Hughston VAS	PROM	1	31	X		
PedsQL	PROM	1	23		X	X

EQ5D-Y, EuroQol five-dimension questionnaire-youth; IKDC, International Knee Documentation Committee; KOOS, Knee injury and Osteoarthritis Outcome Score; KOOS-Child, Knee injury and Osteoarthritis Outcome Score for Children; PROMIS PA-8a, patient-reported outcomes measurement information system paediatric anxiety short form 8 a; PROMIS PDS-8a, patient-reported outcomes measurement information system paediatric depressive symptoms short form 8 a; HSS Pedi-FABS, the Hospital for Special Surgery Paediatric Functional Activity Brief Scale; Pedi-IKDC, Paediatric International Knee Documentation Committee; PedsQL, paediatric quality of life; PROMIS PM-7a, patient-reported outcomes measurement information system paediatric mobility short form 7 a; PROMIS PPA-8a, patient-reported outcomes measurement information system paediatric physical activity short form 8 a; PROM, patient-reported outcome measure; KOOS QOL, Knee Injury and Osteoarthritis Outcome Score Quality of Life subdomain; QoL, quality of life; VAS, visual analogue scale.

## Discussion

Our systematic review highlights a wide coverage and variability of outcomes reported in trials of children and adolescent knee injuries against the WHO ICF outcome domains. These include structure of the lower limb in 40% of primary outcomes and 93.3% of all outcome domains mapped, illustrated in Table III and Table IV. The findings emphasize the critical gaps, inconsistencies and variable reporting in outcome measures, time to outcome, length of follow-up, and outcome selection, further increasing the heterogeneity and reducing the reliability of future meta-analyses. Recognizing these challenges, children’s orthopaedics have led the effort of COS to improve consistent reporting and reduce research waste.^18,20,21,26,34,50^

All trials investigating the outcome of ACL treatment prioritized knee function. The other critical outcomes for ACLs were pain, graft failure, range of motion, and joint stability. For OCD, the critical outcomes were pain, radiological healing (MRI or radiological), quality of life and emotional wellbeing, range of motion, joint stability, and knee function during participation in daily activities. In patella dislocations, all trials prioritized outcomes related to redislocation rate, objective clinical assessment of the patellofemoral morphology, and radiological assessment of the patellofemoral morphology. The other critical outcomes in patella dislocations prioritized outcomes on pain, range of motion, joint stability, and knee function during participation in daily activities. The heterogeneity observed is explained by the individual pathology among different conditions, which is important to highlight as subcategories to consider when developing the core outcome set for children and adolescent knee injuries. Compared to lower limb fractures, joint stability and range of motion are more relevant in knee injuries, which are absent in the COS for children’s limb fractures.^20^

The PROMS used in trials are summarized in Table V, of which only nine (45%) outcome measures were specifically designed for children and adolescents. Inconsistency in using PROMs is reflected in the conflicting and variable outcome domains when mapped against WHO ICF, as demonstrated in Supplementary Table i. The reliability, validity, and responsiveness were assessed across various children’s knee conditions for Pedi-IKDC, developed in 2010.^51^ When compared against KOOS-Child, Pedi-IKDC showed better psychometric properties, as demonstrated in the utility in eight trials (53.3%).^52^ However, the coverage of Pedi-IKDCs against WHO ICF domains was limited, with only ten domains as compared to 17 covered by KOOS and 16 by KOOS-Child. Besides, Pedi-IKDC demonstrated a ceiling effect (in 6% of the study cohort),^51^ especially in domains involving knee function during participation in daily activities, a critical outcome identified in 80% of trials mapped against WHO ICF.

Our study includes limitations. Firstly, strict inclusion criteria were used only to include trials, consistent with previous studies.^19,20^ The search strategies were restricted to studies published in the English language. Although an age group was not specified, the validated paediatric search filters for respective databases were used, followed by a manual screening of age groups of each study at full-text screening. This aims to differentiate paediatric-focused studies on surgical interventions from adult studies that include adolescent age groups. The specific intervention type is described in Supplementary Table ii.

Although this allows us to understand the outcomes perceived to be important in higher-quality level 1 studies, important outcomes that may have only been reported in cohort or case-control studies would may have been excluded, including those in qualitative or narrative studies. Secondly, trials were under-represented internationally. There were representations from North America (n = 6), Europe (n = 5), Australia (n = 2), and China (n = 2), with no representations from low- to middle-income countries. Meniscus pathology was also under-represented, with only a single trial (n = 1). This limits our ability to conclude the outcomes that trialists find important in low- to middle-income countries and for meniscal pathologies.

Our review demonstrates the list of ICF outcome domains reported by trialists that are important in children and adolescent knee injuries, which will guide the development of COS in partnership with other stakeholders: families, patients, allied health professionals, and clinicians.^24,53^ Additionally, our review identified four common outcome domains and mapped them against 35 WHO ICF domains. However, we should consider the views of other stakeholders in other domains during the COS development. From this review, PROMs cannot be recommended for use in specific pathologies, as this study does not measure the outcome measures’ reliability, responsiveness, and validity.

In conclusion, outcomes and outcome measure tools reported in children and adolescent knee injuries are highly variable and inconsistent. Currently, there are no COSs for these injuries, highlighting an urgent need to improve standardization and consistency in trial reporting, including methodologies such as Delphi surveys, COMET methodology, and diverse stakeholders throughout their treatment pathway.^13,17^ A key recommendation for the COS development is accounting for pathology-specific subcategories, given the difference in emphasis on WHO ICF domains across various knee injuries. In the future, these approaches will ensure the COS has comprehensive yet unique priorities for each condition.

**Take home message**

- Outcome measure tools reported in children and adolescent knee injuries are highly variable and inconsistent.

- Currently, there are no core outcome sets (COS) for these injuries, highlighting an urgent need to improve standardization and consistency in trial reporting.

- A key recommendation for the COS development is accounting for pathology-specific subcategories, given the difference in emphasis on WHO ICF domains across various knee injuries.

## Data Availability

The data that support the findings for this study are available to other researchers from the corresponding author upon reasonable request.
